# Characterization and Application of EST-SSR Markers Developed from Transcriptome Sequences in *Elymus breviaristatus* (Poaceae: Triticeae)

**DOI:** 10.3390/genes14020302

**Published:** 2023-01-23

**Authors:** Jin Li, Changbing Zhang, Shiyong Chen, Keke Jiang, Hao Guan, Wenhui Liu

**Affiliations:** 1 College of Animal and Veterinary Sciences, Southwest Minzu University, Chengdu 610041, China; 2 Sichuan Academy of Grassland Science, Chengdu 611731, China; 3 Key Laboratory of Animal Science of State Ethnic Affairs Commission, Southwest Minzu University, Chengdu 610041, China; 4 Key Laboratory of Superior Forage Germplasm in the Qinghai-Tibetan Plateau, Qinghai Academy of Animal Science and Veterinary Medicine, Qinghai University, Xining 810016, China

**Keywords:** *Elymus breviaristatus*, EST-SSRs, transcriptome, genetic diversity, population structure

## Abstract

Background: *Elymus* L. is the largest genus in the Triticeae tribe. Most species in this genus are highly stress resistant, with excellent forage value. *Elymus breviaristatus,* a rare species endemic to the Qinghai-Tibet Plateau (QTP), is declining due to habitat fragmentation. However, genetic data for *E. breviaristatus* are limited, with expressed sequence tag (EST) markers being particularly rare, hampering genetic studies and protection measures. Results: We obtained 9.06 Gb clean sequences from the transcriptome of *E. breviaristatus*, generating 171,522 unigenes, which were assembled and functionally annotated against five public databases. We identified 30,668 SSRs in the *E. breviaristatus* transcriptome, from which 103 EST-SSR primer pairs were randomly selected. Of these, 58 pairs of amplified products of the expected size, and 18 of the amplified products were polymorphic. Model-based Bayesian clustering, the unweighted pair group method with arithmetic average (UPGMA), and principal coordinate analysis (PCoA) of 179 wild *E. breviaristatus* in 12 populations using these EST-SSRs were generally consistent, grouping the 12 populations into two major clades. Analysis of molecular variance (AMOVA) found 70% of the genetic variation among the 12 populations and 30% within the populations, indicating a high level of genetic differentiation (or low gene exchange) among the 12 populations. The transferability of the 58 successful EST-SSR primers to 22 related hexaploid species was 86.2–98.3%. UPGMA analysis generally grouped species with similar genome types together. Conclusions: Here, we developed EST-SSR markers from the transcriptome of *E. breviaristatus.* The transferability of these markers was evaluated, and the genetic structure and diversity of *E. breviaristatus* were explored. Our results provide a basis for the conservation and management of this endangered species, and the obtained molecular markers represent valuable resources for the exploration of genetic relationships among species in the *Elymus* genus.

## 1. Introduction

*Elymus* sp. is an economically important genus in the grass tribe Triticeae in the family Poaceae, which is the largest genus in the Triticeae and contains more than 150 species worldwide [1]. Most species in this genus are widely distributed in temperate regions and have excellent resistance to disease, drought, and cold [2]. Therefore, *Elymus* represents an indispensable genetic resource for the improvement of stress tolerance in related crops and forage grass [3]. *Elymus breviaristatus* (Keng) Keng f. is a bunch-type, self-pollinating, short-lived, perennial forage grass with an StStHHYY genome (2n = 6*x* = 42) endemic to the Qinghai-Tibet Plateau (QTP) [4]. Because of its strong adaptability and high feeding value, *E. breviaristatus* is considered an excellent forage grass in the alpine region, which plays an important role in grassland husbandry and ecological restoration on the QTP [5]. However, due to increased human activity and habitat deterioration on the QTP, the wild populations of *E. breviaristatus* have been significantly reducing over the past years, and it has been listed as an important conserved wild plant in China (Class II) since 1999. Therefore, it is critical to accelerate the conservation and utilization of *E. breviaristatus*, particularly in revealing the distribution and genetic diversity of wild populations.

In recent decades, many types of molecular markers have been widely used in species diversity assessments, molecular-assisted breeding, DNA fingerprinting, and conservation biology [6,7,8]. Simple sequence repeats (SSRs) markers tend to be commonly used because of their high polymorphism, co-dominant inheritance, wide genome coverage, and good reproducibility [9,10,11]. In particular, expressed sequence tag SSRs (EST-SSRs) show more convenient development and higher transferability across related species than genomic SSRs, which have a wide range of applications in population genetics and breeding of crop and forage grass [12], especially in non-model species. However, owing to a lack of genetic and genomic information, there have been no investigations of EST-SSR markers in *E. breviaristatus*. Although previous studies have used the EST-SSRs of related *Elymus* species and SRAPs to analyze the genetic structure of *E. breviaristatus* [13,14], the number of markers was too small, and detection efficiencies remain to be improved. Thus, the more and higher efficient markers are needed for an in-depth understanding of the diversity of *E. breviaristatus* and to develop strategies for their sustainable utilization.

As next-generation sequencing techniques have become more advanced, several new methods of SSR marker development have been reported [15,16]. For example, de novo transcriptome sequencing is an advanced high-throughput sequencing technique that can generate transcriptome sequences containing large numbers of EST-SSRs within a short timeframe [17]. EST-SSRs developed from the transcriptional regions of the genome are evolutionarily conserved and have a high level of transferability to related species [18]. However, to date, the transcriptome of *E. breviaristatus* remains unavailable, which has hindered critical aspects of research into this species, such as protection management and the molecular mechanisms underlying its desirable traits. Here, we sequenced the transcriptome of *E. breviaristatus* using the Illumina Hiseq platform, and then assembled and annotated the sequence data, which was the first systematic report of the transcriptome of *E. breviaristatus*. Based on these data, we identified EST-SSR loci and developed primer pairs. The major objectives of this study were to develop efficient EST-SSR markers for genetic diversity analyses of the endangered plant *E. breviaristatus* and to assess the applicability of these markers to related species.

## 2. Materials and Methods

### 2.1. Plant Materials

**Figure 1:**
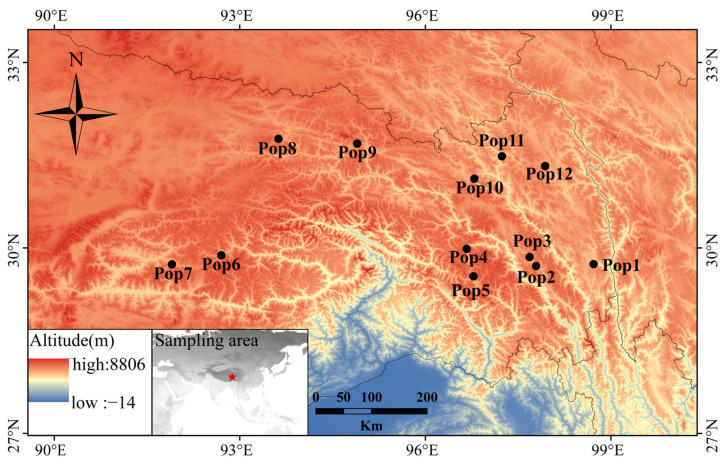
Geographic distribution of the 12 *E. breviaristatus* populations used in this study.

The leaves used for RNA isolation and transcriptome sequencing were collected from a single individual of *E. breviaristatus* cv. Chuanxi growing in Hongyuan county, China (latitude, 32.78 °N; longitude, 102.54 °E; altitude, 3502 m). For the genetic diversity analysis, we collected 179 *E. breviaristatus* individuals from 12 locations on the Qinghai-Tibet Plateau (Figure 1, Table S1). In each of the 12 locations, we collected samples from 14–15 individual plants that were a minimum of 5 m apart. In addition, a total of 23 related allohexaploid species of Elymus and Roegneria in Triticeae were used to test the cross-species transferability of the identified EST-SSRs (Table S2). All materials in the study were planted in the forage grass nursery of Southwest Minzu University (Hongyuan, Sichuan, China).

### 2.2. RNA Sequencing, Transcriptome Assembly, and Transcriptome Annotation

RNA was isolated using the Plant Total RNA Kit (Tiangen Biotech Co., Ltd., Beijing, China), following the manufacturer’s instructions. RNA quality was verified using an Agilent Bioanalyzer 2100 system (Agilent Technologies, Palo Alto, CA, USA). RNA degradation and contamination were monitored on 1% agarose gels. Total RNA purity was checked using a NanoPhotometer spectrophotometer (Implen, Calabasas, CA, USA). Sequencing libraries were generated using a NEBNext Ultra RNA Library Prep Kit for Illumina (NEB, San Diego, CA, USA), following the manufacturer’s instructions, and were preliminarily quantified using a Qubit2.0 fluorometer (Life Technologies, Carlsbad, CA, USA). The insert size of the library was determined using the Agilent Bioanalyzer 2100. The library preparations were performed on the Illumina Hiseq 2500 platform.

The sequenced raw reads were cleaned by removing adapter sequences and low-quality reads. De novo transcriptome assembly of the clean reads was performed using Trinity [19]. The assembled unigene sequences were searched against several public databases, including Gene Ontology (GO) [20], Clusters of Orthologous Groups of proteins (COG) [21], euKaryotic Orthologous Groups (KOG), and Kyoto Encyclopedia of Genes and Genomes (KEGG) [22,23]. Unigenes were functionally annotated by first searching for homologous sequences in the NCBI non-redundant protein sequence (NR) database using BLAST (E-value < 10^–5^) [24], and then searching the resulting sequence hits against the Protein family (Pfam) database using HMMER to obtain annotation information [25].

### 2.3. EST-SSR Marker Identification and Primer Design

SSRs were identified in the assembled unigene sequences using the MicroSatellite (MISA) identification tool [26]; the minimum numbers of repeats for mono-, di-, tri-, tetra-, penta-, and hexa-nucleotide motifs were set to 10, 6, 5, 5, 5, and 5, respectively. The EST-SSR primers were designed using Primer 3 (http://primer3.sourceforge.net, accessed on 15 March 2020) based on the MISA results. From the designed EST-SSR primers, we randomly selected 103 pairs for synthesis using Tingke Biological Technology (Beijing, China).

### 2.4. DNA Extraction and EST-SSR Amplification

Fresh young leaves were collected from the two sets of plant materials described above and dried in zip-lock plastic bags with silica gel. Total genomic DNA was extracted using a DP350 Plant DNA kit (Tiangen Biotech Co., Ltd., Beijing, China), following the manufacturer’s instructions. The quality and quantity of DNA were detected using 1% agarose gels and a NanoDrop-Lite spectrophotometer (Thermo Scientific, Waltham, MA, USA), respectively. DNA samples were diluted to a concentration of 10 ng/μL and stored at −20 °C.

PCR amplifications were performed in a 20-μL reaction volume containing 20 ng of genomic DNA, 0.5 μM of each primer, and 10 μL of 2×Es Taq MasterMix (Dye Plus) (CoWin Biosciences, Beijing, China). A PTC-200 Thermal Cycler (BIO-RAD, CA, United States) was used to run touch-down PCRs with the following cycling conditions: 1 cycle of 94 °C for 4 min; 5 cycles of 94 °C for 30 s, 60–65 °C for 30 s, and 72 °C for 60 s; 35 cycles of 94 °C for 30 s, 60 °C for 30 s, and 72 °C for 60 s; 1 cycle of 72 °C for 10 min; and an indefinite hold at 4 °C. The PCR products were separated using 6% non-denaturing polyacrylamide gel electrophoresis (PAGE). Products were loaded into the gel in 1×TBE (Tris-borate-EDTA) buffer and run at 400 V for 1.5 h, along with a 100 bp molecular size ladder (Tiangen Biotech Co., Ltd., Beijing, China). Bands were then visualized using silver staining.

### 2.5. Statistical Analyses

The EST-SSR profiles obtained for each sample were scored as present (1) or absent (0), and transformed into a raw data matrix. Using this data matrix, we calculated several genetic diversity parameters among the 12 populations with POPGENE1.32 [27]: percentage of polymorphic bands (PPB), Nei’s genetic diversity (H), Shannon information index (I), observed number of alleles (Na), number of effective alleles (Ne), and Nei’s genetic distance (GD). The PIC was calculated for each primer as “PIC” = 2f(1−f), where f is the amplified allele frequency and 1−f is the frequency of the null allele [28]. Matrixes showing Nei’s pairwise genetic distances for 179 *E. breviaristatus* individuals and 23 related species were calculated using NTSYSpc 2.10e [29]. Dendrograms were constructed using the unweighted pair-group method with an arithmetic mean (UPGMA) algorithm in MEGA 6.0 based on Nei’s genetic distances [30]. Principal coordinate analysis (PCoA) and analysis of molecular variance (AMOVA) of the EST-SSRs were performed using GenAlEx 6.5102 [31].

A model-based Bayesian clustering approach in STRUCTURE v.2.3.4 [32] was used to analyze the genetic structure across 179 individuals and to determine the most likely number of clusters (K). For each value of K from 2 to 12, 20 independent runs were performed. Each run comprised 50,000 Markov chain Monte Carlo (MCMC) replicates with an admixture. The first 10,000 replicates were discarded as burn-in. The optimum K value was estimated using Structure Harverster [33] based on Ln P (D) and delta K. Finally, the 10 replicates were clustered and aligned using CLUMPAK [34].

## 3. Results

### 3.1. Illumina Sequencing and De Novo Transcriptome Assembly

A total of 61,494,104 paired-end raw sequencing reads were generated and submitted to NCBI’s SRA under accession number SRR16939514, after filtering and strict quality checking, 60,407,780 clean reads (9.06 Gb) remained. Across the clean reads, the Q20 and Q30 ratios were over 96% and 91%, respectively. The GC content of the clean reads was 57.08%. A total of 250,182 transcripts were assembled, with an average length of 860 bp. These transcripts represented 171,522 unigenes, with a total length of 187,855,868 bp, an average length of 1095 bp, an N50 of 1409 bp, and an N90 value of 570 bp (Table 1).

### 3.2. Unigene Annotation

Of the 171,522 identified unigenes, 153,008 (89.2%) were successfully annotated in at least one of the searched databases (NR, NT, GO, KEGG, KOG, Swiss-Prot, and Pfam), and 17,055 (9.94%) were annotated in all eight databases. Specifically, 124,901 (72.82%) were annotated in NR; 140,346 (81.82%) were annotated in NT; 42,822 (24.69%) were annotated in KEGG; 80,171 (46.74%) were annotated in Swiss-Prot; 84,222 (49.10%) were annotated in Pfam; 85,556 (49.88%) were annotated in GO; and 27,850 (16.23%) were annotated in KOG.

**Figure 2:**
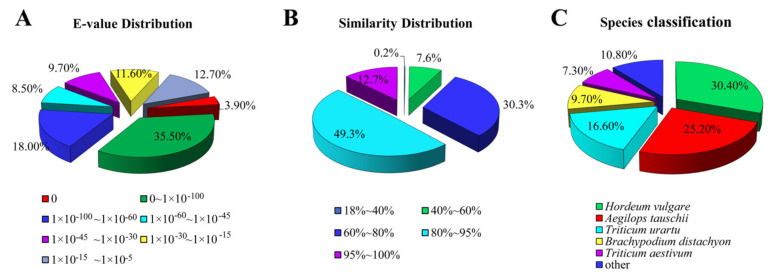
Distributions of unigene annotations against the NR database: (**A**) E-value distribution, (**B**) similarity distribution, and (**C**) species distribution.

The E-value distributions of the NR annotations indicated that 39.40% of the unigenes yielded significant hits (Figure 2A), while approximately 62.00% of the unigene sequences exhibited a greater than 80% identity with the other sequences (Figure 2B). BLAST homology searches showed that five species with the highest overall sequence similarities to *E. breviaristatus* unigene sequences in the NR database were *Hordeum vulgare* (30.4%), *Aegilops tauschii* (25.2%), *Triticum urartu* (16.6%), *Brachypodium distachyon* (9.7%), and *Triticum aestivum* (7.3%) (Figure 2C).

**Figure 3:**
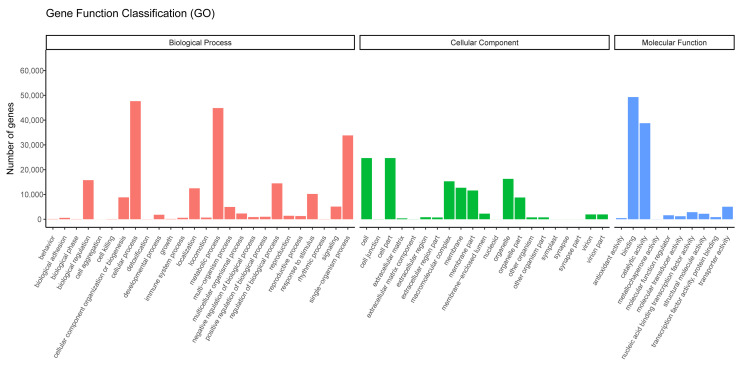
Gene Ontology terms enriched in the assembled unigenes.

We successfully annotated 85,556 unigenes against the GO database. The terms enriched in the unigenes represented 57 functional sub-groups within the three main GO groups: biological process (209,312 unigenes; 48.07%), cellular component (123,695 unigenes; 28.41%), and molecular function (102,405 unigenes; 23.52%; Figure 3). In the biological process category, the terms most commonly enriched in the unigenes were “cellular processes” (44,860 unigenes; 21.43%), and “single-organism processes” (33,833 unigenes; 16.16%), and in the cellular component category, the terms most commonly enriched in the unigenes were “cell” (24,676 unigenes; 19.95%), “cell part” (24,679 unigenes; 19.95%), “organelle” (16,300 unigenes; 13.18%), and “macromolecular complex” (15,309 unigenes; 12.38%; Figure 3). Finally, in the molecular function category, the terms most commonly enriched in the unigenes were “binding” (49,290 unigenes; 48.13%) and “catalytic activity” (38,772 unigenes; 37.86%; Figure 3).

**Figure 4:**
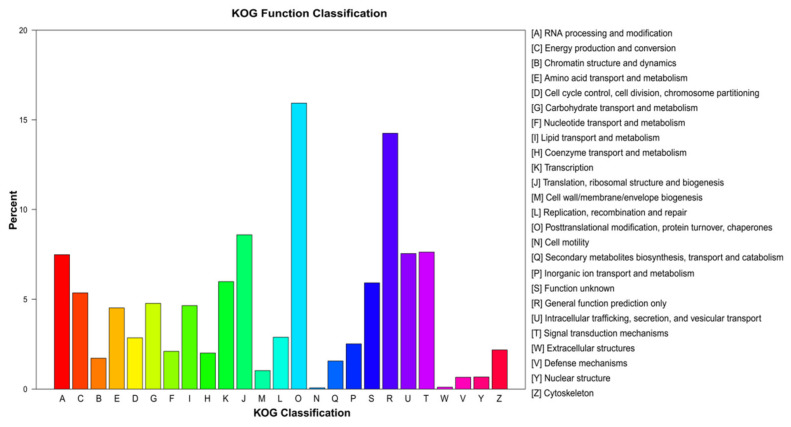
euKaryotic Orthologous Groups analysis of the unigene sequences of *E. breviaristatus*.

We successfully assigned 27,850 unigenes to 25 KOG functional categories. The category assigned the most unigenes was “posttranslational modification, protein turnover, chaperones” (4437 unigenes; 15.93%), followed by “general function prediction only” (3936 unigenes; 14.25%), and “translation, ribosomal structure, and biogenesis” (2392 unigenes; 8.59%; Figure 4). The least well-represented KOG categories were “extracellular structures” (30 unigenes; 0.11%) and “cell motility” (19 unigenes; 0.07%; Figure 4).

**Figure 5:**
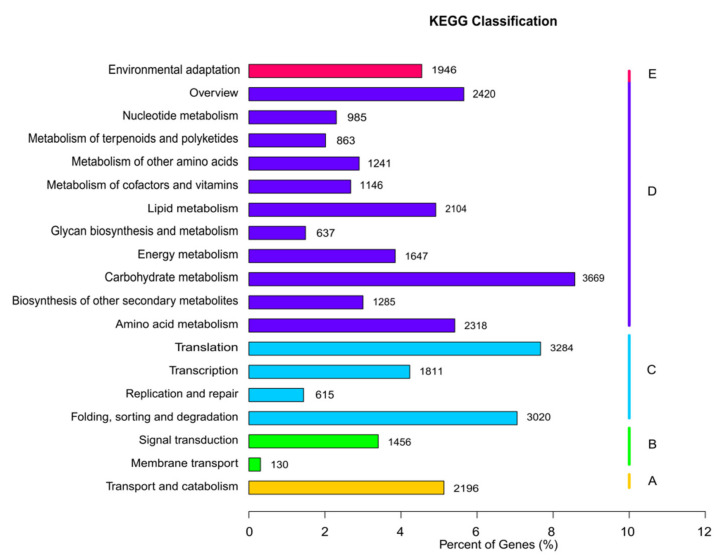
Kyoto Encyclopedia of Genes and Genomes analysis showing clusters of orthologous groups.

A total of 42,882 unigenes were associated with 129 KEGG pathways in five main categories: organismal systems, metabolism, genetic information processing, environmental information processing, and cellular processes (Figure 5). The category associated with the most unigenes was metabolism, which included 18,315 unigenes distributed among 99 KEGG pathways, followed by genetic information processing, which included 8730 unigenes distributed among 21 KEGG pathways. Only 5728 unigenes were associated with the remaining three categories (organismal systems, environmental information processing, and cellular processes). Across all categories, the three KEGG pathways associated with the most unigenes were “carbohydrate metabolism” (3669 unigenes; 8.57%), “translation” (3284 unigenes; 7.67%), and “folding, sorting, and degradation” (3020 unigenes; 7.05%).

### 3.3. Distribution and Frequency of SSR Markers

To develop new SSRs, we mined microsatellites from 171,522 unigenes. We identified 30,668 SSRs in the 25,834 SSR-containing sequences; 3973 sequences contained more than one EST-SSR, and 2054 of these were compound SSRs. Across the 30,668 identified SSRs, trinucleotide repeats were the most common (14,416 SSRs; 47.01%), followed by mononucleotide repeats (9096 SSRs; 29.66%), and dinucleotide repeats (6241 SSRs; 20.35%); other types of SSRs were rare (Table 2). Across the mono- to hexanucleotide repeats (Table S3), the most common repeat motifs were A/T (25.32%), AG/CT (11.75%), CCG/CGG (18.99%), ACGC/CGTG or AGGG/CCCT (9.56%), AAGGG/CCCTT (9.09%), and AGCAGG/CCTGCT (12.5%). The EST-SSRs most commonly included five repeat motifs (9365 EST-SSRs; 30.54%), followed by six repeat motifs (5995 EST-SSRs; 19.55%), ten repeat motifs (3997 EST-SSRs; 13.03%), and seven repeat motifs (2351 EST-SSRs; 7.67%).

### 3.4. Development and Validation of SSR Markers

**Figure 6:**
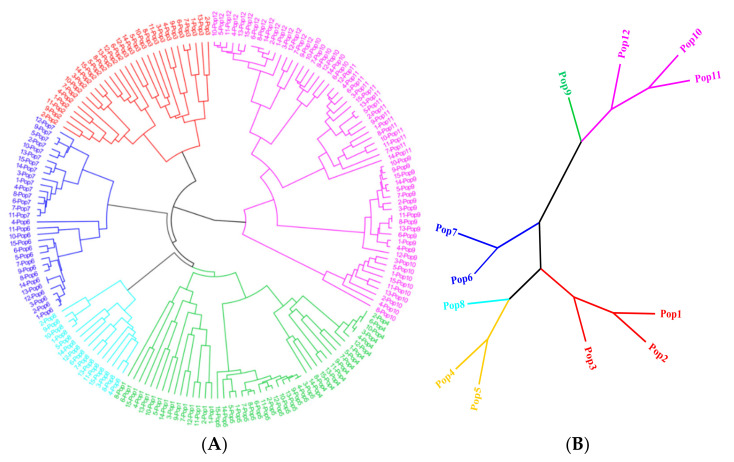
UPGMA dendrogram showing (**A**) 179 *E. breviaristatus* individuals and (**B**) 12 *E. breviaristatus* populations based on pairwise genetic distances.

From all SSR primer pairs, 103 pairs of primers were randomly selected and used to amplify SSRs from the DNA of 12 geographically distinct *E. breviaristatus* populations. In total, 58 primer pairs amplified PCR products of the expected size (Table S3), and 18 of these amplified SSRs were polymorphic (Table 3). We used the 18 polymorphic primer pairs identified above to investigate the relationships among wild populations and individuals of *E. breviaristatus*. This analysis generated 164 consistently well-amplified bands (an average of 9.1 bands per primer), among which 146 were polymorphic. The polymorphic information content (PIC) for the 18 primers was 0.267–0.500, with an average of 0.457. The mean of Nei’s genetic diversity (H) and Shannon information index (I) ranged from 0.155–0.410 and 0.250–0.588, respectively (Table 3). The UPGMA dendrogram for the 179 *E. breviaristatus* individuals recovered the samples in two major clades, with one clade including one subclade and the other clade including five subclades (Figure 6A). Across the five subclades, all populations were monophyletic, except for population 10, which was polyphyletic (although still in the same subclade; Figure 6A).

We assessed several indexes of α diversity at the population level. Across all populations, the mean number of alleles (Na) was 5.611–12.000, the mean number of expected alleles (Ne) was 9.513–11.400, the Shannon information index (I) was 0.043–0.237, expected heterozygosity (He) was 0.028–0.153, and unbiased heterozygosity (uHe) was 0.028–0.159 (Table 4).

In all indexes, diversity was lowest in Population 5 and highest in Population 1. Pairwise genetic distances ranged from 0.107 (between Population 1 and Population 2) to 0.462 (between Population 5 and Population 9). The UPGMA dendrogram for the 12 populations showed them divided into two groups, which showed that group I contained populations 1–8, and group II contained populations 9–12 (Figure 6B). Finally, the analysis of molecular variance (AMOVA) of the EST-SSRs showed that 70% of the genetic variation existed among populations, while only 30% of the genetic variation existed within populations.

### 3.5. Population Genetic Structure of E. breviaristatus

**Figure 7:**
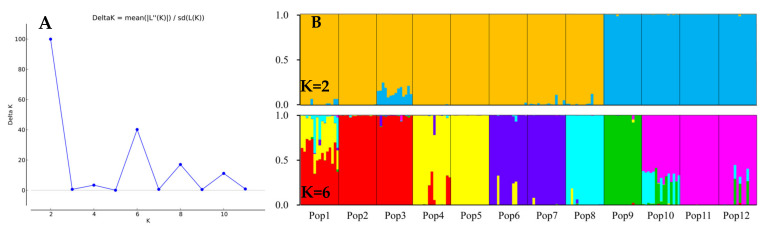
Genetic structure of the 12 populations of *E. breviaristatus* estimated with 18 EST-SSR markers. (**A**) Delta K of all values of K from 2–12. (**B**) Vertical bars represent the 12 *E. breviaristatus* populations for K = 2 and K = 6, and bars are divided into several colors when there is evidence of admixture.

Using a model-based Bayesian clustering approach, we analyzed the genetic structure among the *E. breviaristatus* individuals and found that ΔK was maximized at K = 2 (Figure 7A). This indicated that the 179 individuals likely comprised two genetic sub-populations: one sub-population included populations 9–12, while the other sub-population included populations 1–8 (Figure 7B). At K = 6, which represented the next highest value of ΔK (Figure 7B), six sub-populations were grouped as follows: Populations 1–3, Populations 4–5, Population 6–7, Population 8, Population 9, and Population 10–12 (Figure 7B). These results were consistent with the population-level UPGMA dendrogram (Figure 6B).

**Figure 8:**
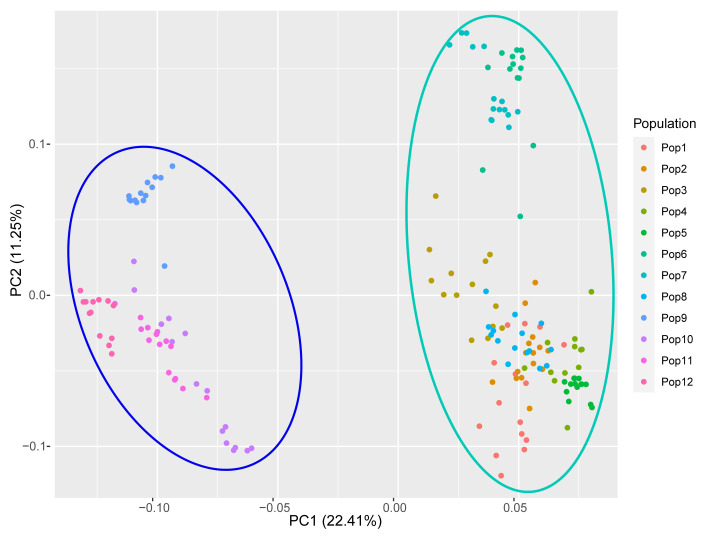
Principal coordinate analysis (PCoA) of the 12 populations of *E. breviaristatus* based on genotypic information from 18 EST-SSR markers.

Principal coordinate analysis (PCoA) recovered the 12 populations in two major groups (Figure 8); the first two principal coordinates explained 33.56% of the total genetic variance. These groups were consistent with those recovered by the clustering approach when K = 2 (Figure 7B), and fairly consistent with those recovered by the population-level UPGMA analysis (Figure 6B).

### 3.6. Transferability of EST-SSRs to Related Species

**Figure 9:**
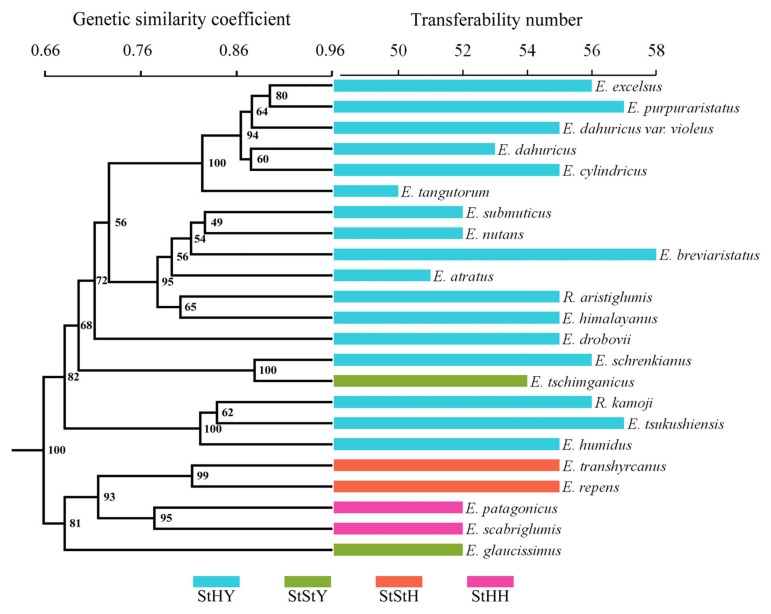
Degree of EST-SSR marker transferability to 22 species closely related to *E. breviaristatus* mapped onto a UPGMA dendrogram.

To evaluate the transferability of the EST-SSRs from *E. breviaristatus*, we performed cross-species amplification analysis of these 58 primer pairs in the Triticeae species with StHY, StStH, StHH, and StStY genomes. Transferability was greatest in *E. purpuraristatus* (57 successful EST-SSRs; 98.3%) and lowest in *E. tangutorum* (50 successful EST-SSRs; 86.2%). The overall transferability rate was 91.2%. Among the 23 species, a total of 391 reliable bands were generated, of which 369 bands (94.37%) were polymorphic. The Dice genetic similarities (GS) of 23 species ranged from 0.551 (*E. tangutorum* and *E. repens*)–0.915 (*E. excelsus* and *E. purpuraristatus*), which showed a high level of genetic variation among the species. The dendrogram constructed based on Dice GS values with UPGMA analysis obviously divided the 23 accessions into two groups (100% bootstrap support), which showed that the species with the same or similar genomes could be grouped together (Figure 9). Cluster I was comprised of *E. patagonicus* (StHH), *E. scabriglumis* (StHH), *E. repens* (StStH), *E. transhyrcanus* (StStH), and *E. glaucissimus* (StStY). Cluster II mainly contained all the StYH genome species except one StStY species, *E. tschimganicus*.

## 4. Discussion

### 4.1. Characterization of SSRs in the Transcriptome

As next-generation sequencing has become increasingly accurate, rapid, and inexpensive, SSR markers have been developed for a number of species. At present, SSRs have been used to explore genetic diversity and population structure in several *Elymus* species [35,36,37]. However, to date, there are almost no available EST sequences for *E. breviaristatus*. Here, we provide the first report of the *E. breviaristatus* transcriptome, which was generated using next-generation sequencing. In total, 60,407,780 clean reads with a Q20 of 96.59% were obtained from 61,494,104 paired-end raw reads, and 171,522 unigenes were identified in the assembled transcriptome with a mean length of 1095 bp and an N50 of 1409 bp, which ensured the quality of sequencing. The N50 values of unigenes were consistent with the results reported in *Glycyrrhiza uralensis* (1395 bp) [38], radish (*Raphanus sativus*) (1256 bp) [39], and common bean (1449 bp) [40]. Overall, the transcriptome assembly data of *E. breviaristatus* generated in the present study will be valuable for use in subsequent analyses.

From the 25,834 SSR-containing sequences in the *E. breviaristatus* transcriptome, we identified 30,668 SSRs. The most common motifs were trinucleotide repeats (14,416 SSRs; 47.01%), mononucleotide repeats (9.096 SSRs; 29.66%), and dinucleotide repeats (6241 SSRs; 20.35%). Similar patterns of SSR motif abundance have been found in most other plant genomes, such as *E. nutans* [41] and alfalfa [42], showing that the trinucleotide repeat is the most common motif (64.27% and 48.80%, respectively). Previous studies have suggested that the most abundant trinucleotide repetition may be affected by harmful mutations in translated regions, resulting in the inhibition of the expansion or contraction of dinucleotide repeats in exons [43]. As shown in Table S4, among all types of sequence motifs identified in the *E. breviaristatus*, excluding the mononucleotide repeats, the CCG/CGG motif was the most common (5823 SSR), followed by AG/CT (3602 SSRs), AGG/CCT (2548 SSRs), and AGC/CTG (2258 SSRs). These results were consistent with patterns of sequence motifs found in the congeneric *E. nutans* [41] but differed from patterns of sequence motifs found in dicotyledonous species, such as *Medicago truncatula* [44], *Ipomoea batatas* [45], and *Menispermum dauricum* [46]. This reflects the difference between monocotyledons and dicotyledons. Previous studies showed that the CCG/CGG was the most abundant motif in monocotyledons but a rare repeat type in dicotyledons [47,48].

Of the 103 randomly selected SSR primer pairs, 58 successfully amplified fragments from *E. breviaristatus* DNA, and 18 of these fragments were polymorphic. This PCR success rate (56.31%) was higher than that reported for tree peony (47.30%) [49] and alfalfa (30%) [42]. However, the percentage of EST-SSR polymorphic markers in the present study was 17.48%, which did not reach the rate of the above species (39.90% and 29%). The differences in PCR success rates among these species might be due to differences in ploidy, while the level of polymorphism may be limited by the sample size and geographical origin of the materials used [50]. In general, the genetic data obtained in the study compensate for the lack of a reference genome for *E. breviaristatus,* and our transcriptome sequencing data will support novel gene discovery or the investigation of molecular mechanisms in this species and related taxa.

### 4.2. Genetic Diversity and Structure of E. breviarstatus Populations

Based on the amplification results of the 18 polymorphic EST-SSR primers, the percentage of polymorphic bands and the average PIC value were higher than those of a previous study of *E. breviaristatus*, which developed EST-SSR markers from *E. wawawaiensis and E. lanceolatus* [13], indicating that our primers might be more suitable for studies of genetic diversity and population structure. However, genetic diversity analyses of the 12 *E. breviaristatus* populations showed that the Shannon index (I) across the populations was 0.127, which is lower than that reported in other *Elymus* species, including *E. sibiricus* (0.297) and *E. nutans* (0.344) [15]. Previous studies have similarly shown that genetic diversity among and within *E. breviaristatus* populations is low [14]. This low level of population genetic diversity was unsurprising, as *E. breviaristatus* is a rare species endemic to QTP, and its genetic diversity may be restricted by geographical scope, environmental factors, and mating systems [51].

UPGMA cluster analysis, PCoA analysis, and Bayesian population structure analysis suggested that 179 *E. breviaristatus* accessions could be divided into two major clades, which revealed an obvious pattern of genetic differentiation between the major groups of *E. breviaristatus* populations. This consistency suggests that our results were reliable. Meanwhile, the AMOVA showed that genetic variation mainly occurred among populations rather than within populations. Similar results have been reported for several other self-pollinating *Elymus* species, including *E. canadensis* [52], *E. fibrosus* [53], and *E. caninus* [54]. Compared to the results of previous studies [13,14], the genetic variation within *E. breviaristatus* populations in our study area was extremely low, which may be due to the complex environment of the Qinghai-Tibet Plateau leading to multiple genetic differentiation mechanisms of the same species in different regions [55]. In general, our results will be useful for developing protection management plans for this species, such as in situ conservation strategies that limit grazing and reduce human disturbance to protect existing populations with high levels of genetic diversity [51,56], while ex situ conservation strategies can establish a diversified genetic resource bank of *E. breviaristatus* by collecting representative materials from populations with high genetic differentiation [13].

### 4.3. Transferability of EST-SSRs and Phylogenetic Relationships of Elymus Species

Due to the conservative nature of the flanking sequences of repeat loci, the SSR markers showed transferability in related species. Generally, the EST-SSRs have a higher transferability than other molecular markers on account of the conservation of transcribed regions among related species [12]. In the study, we also explored the transferability of the 58 successful EST-SSRs to 22 related species. In each of the related species, 86.2–98.3% of the primer pairs were successful, which showed higher transferability in the hexaploid species of *Elymus* and *Roegneria*. The transferability of these EST-SSRs was similar to the transferability ratios observed in *E. wawawaiensis* (96.08%) [35] but higher than that observed in *E. sibiricus* (49.11%) [57]. The different transferability rates may be influenced by the phylogenetic relationships between species. Usually, closely related species or species with high genomic similarity have high marker transferability [15]. In this study, the high transferability of these primers among other *Elymus* species indicates that these primers represent a valuable resource for the development of molecular markers for *Elymus* species.

As the largest genus in Triticeae, the taxonomy and biosystematics of *Elymus* species are extremely complex because of the huge morphological variation, the polyploid origin, and spontaneous hybridizations among the species [2], which is also one of the essential theoretical bases for their utilization. There has been a lot of work done on the phylogenetic relationships of tetraploid species with StH and StY genome constitution, which revealed the origin of the species and the reticulate evolutionary relationships. However, there are only a few studies on the biosystematic relationships of hexaploidy species, especially in StHY species. In this study, the phylogenetic relationships of the hexaploidy species with St, H, and Y genomes were revealed based on SSR data, which showed that the species with the same or similar genomes could be grouped together. The species with the StYH genome were clustered together and showed a relatively distant affinity with other StYY, StStY, StStH, and StHH species. Then, three different variation types were also revealed among the species with the StHY genome, which was also consistent with the morphology. These results indicate that the markers developed in this study would provide a powerful molecular tool for evolutionary adaptation and genetic relationship analysis in *Elymus*. In addition, the genetic information revealed by these primers can help to determine phylogenetic relationships in the *Elymus*, particularly if combined with further evidence, such as additional gene sequences and/or cytological [58] or morphological [59] characteristics.

## 5. Conclusions

In this study, we were the first to successfully develop 58 EST-SSR markers from the transcriptome of *E. breviaristatus*, 18 of which were polymorphic. Based on the amplification results of the polymorphic primers. The UPGMA, PCA, and structure analysis support that the genetic background of *E. breviaristatus* populations can be divided into two categories, which is accompanied by a low level of genetic diversity (mean I = 0.127) and genetic variation among populations was dominant (70%). These genetic characteristics can provide an important basis for further resource evaluation, collection, and conservation. In addition, 58 EST-SSR markers that can be successfully amplified have excellent transferability in related species (86.2% to 98.3%) and can classify most species according to their genome types through the amplification results, which provides valuable resources for exploring the genetic relationship among species in the *Elymus* genus.

## Figures and Tables

**Table 1:** Overview of de novo sequence assembly for *E. breviaristatus*.

Length Range (bp)	Transcripts	Unigene
200–500	106,453	36,753
500–1000	67,045	59,577
1000–2000	57,272	55,935
>2000	19,412	19,257
Total Number	250,182	171,522
Total Length	215,188,383	187,855,868
N50 Length	1268	1409
N90 Length	377	570
Mean Length	860	1095

**Table 2:** The distribution of EST-SSRs based on the number of repeat units.

Number of Repeat Units	Mono-	Di-	Tri-	Tetra-	Penta-	Hexa-	Total	Percentage (%)
5	0	0	8687	505	119	54	9365	30.54
6	0	2263	3544	166	5	17	5995	19.55
7	0	961	1360	22	7	1	2351	7.67
8	0	668	560	12	1	0	1241	4.05
9	0	537	88	3	0	0	628	2.05
10	3616	285	96	0	0	0	3997	13.03
11	1460	300	27	1	0	0	1788	5.83
12	840	307	17	2	0	0	1166	3.80
13	481	107	22	0	0	0	610	1.99
14	402	126	5	0	0	0	533	1.74
15	332	138	1	0	0	0	471	1.54
>15	1965	549	9	0	0	0	2797	9.12
Total	9096	6241	14,416	711	132	72	30,668	100
Percentage (%)	29.66	20.35	47.01	2.32	0.43	0.23	100	

**Table 3:** Characterization of 18 polymorphic primer pairs in *E. breviaristatus*.

Primers	Primer Sequence (5′-3′)	Repeat Motif	Fragment Size (bp)	PIC	Mean H	Mean I
MDEB002	F: AGTCCTTAGCCCCTAGGACG	(CT) 9	204–246	0.499	0.271	0.422
	R: AAAGAGAGGAGGGCTGGGAT					
MDEB024	F: ATGTATGGGGCGGGCATATG	(CAT) 5	203–224	0.500	0.125	0.208
	R: CAAACCGACGAGAGGTGTCA					
MDEB025	F: GATCCGACGACCTCAGCTTC	(CCA) 5	197–228	0.487	0.316	0.466
	R: CCATCTGCAGCTGGTCAAGA					
MDEB027	F: CATCGACATCACCTGGGTGT	(CCG) 5	227–241	0.498	0.275	0.414
	R: TTCTTGTTCCCCACGGCTTC					
MDEB046	F: TACATCAAAGCGCAGGCAGA	(CTA) 6	176–267	0.497	0.316	0.469
	R: AAGGAGTTGACATGGCTCGG					
MDEB050	F: CGCCTACACAAGATGGTGGT	(CTG) 5	172–257	0.499	0.425	0.612
	R: CACTCTGTCGAAATGCAGCG					
MDEB051	F: GCCTGCTGAAGATGCTCAGA	(CTG) 5	148–237	0.451	0.387	0.571
	R: CCAGCAGTACAACCAGAGCA					
MDEB054	F: ACCGGCATTTCCTACACTGG	(GAC) 6	226–235	0.419	0.304	0.475
	R: TCCTCACAGCTCTCACCACA					
MDEB063	F: CACTTCTCCCCTCGCGATG	(GCG) 5	240–277	0.500	0.410	0.588
	R: AACATGAGCAGGCTCTCGAC					
MDEB065	F: CAAGAGCGACCTGGTGAAGT	(GCG) 5	156–230	0.469	0.316	0.481
	R: CTCGTTCCCATCATCCGCAA					
MDEB067	F: AATGGTTCGTTGTCGTCGGA	(GCG) 6	211–219	0.496	0.301	0.453
	R: GTGGTGGACAAGTAGCTGCT					
MDEB068	F: GTAACTGTTCTTCGGCGGGA	(GCT) 5	145–231	0.399	0.363	0.530
	R: CCACCGAAGAACGACTACCC					
MDEB076	F: ATCCACGTTTCCCTCTGCTG	(GGC) 5	229–248	0.487	0.400	0.586
	R: CCAGTCCCCACTACCAGCTA					
MDEB083	F: TGAGGCTGGGACTGAAGAGT	(GTC) 5	191–212	0.443	0.239	0.335
	R: CTTCTCAGCTGCTGACCTCC					
MDEB091	F: ACCCCCAGCTACTACCATGT	(TCA) 6	226–248	0.267	0.155	0.250
	R: GAGATGGAGCCTACCGAAGC					
MDEB094	F: CGCCTCTTCCACGTCTTTGA	(TCG) 5	138–267	0.493	0.294	0.443
	R: GATGGTGCTCCTCGAGATCG					
MDEB095	F: CAGCTGTTCTGGAGGGTGAG	(TCT) 5	216–280	0.355	0.162	0.258
	R: TCAGAAGGTGACGACGCTTC					
MDEB100	F: TGGTCGCGAGATCTTATGGG	(TGT) 5	184–193	0.475	0.248	0.411
	R: TAAACAACAGCACCTGGCCT					

Note: H = Nei’s genetic diversity; I = Shannon information index.

**Table 4:** Genetic parameters of the 12 populations of *E. breviaristatus*.

Pop ID	N	Na	Ne	I	He	uHe
Pop1	15	12.000	11.400	0.237	0.153	0.159
Pop2	15	8.167	10.662	0.143	0.097	0.100
Pop3	14	9.000	10.965	0.175	0.118	0.123
Pop4	15	8.500	10.754	0.154	0.104	0.107
Pop5	15	5.611	9.513	0.043	0.028	0.028
Pop6	15	7.667	10.246	0.111	0.074	0.077
Pop7	15	6.389	9.950	0.081	0.054	0.056
Pop8	15	8.889	10.837	0.164	0.110	0.114
Pop9	15	6.333	9.733	0.059	0.039	0.041
Pop10	15	8.389	10.902	0.175	0.117	0.121
Pop11	15	7.167	10.202	0.107	0.071	0.073
Pop12	15	6.444	9.881	0.078	0.050	0.052
Mean		7.880	10.42	0.127	0.085	0.088

Note: N = number of individuals; Na = mean number of alleles; Ne = mean number of expected alleles; I = Shannon information index; He = expected heterozygosity; uHe = unbiased heterozygosity.

## Data Availability

We stored the raw sequence in the Sequence Read Archive of the National Center for Biotechnology Information (NCBI), and they are identified with the SRA under the accession number SRR16939514.

## Supplementary Materials

The following supporting information can be downloaded at: https://www.mdpi.com/article/10.3390/genes14020302/s1, Table S1: Geographic information of the 12 *E. breviaristatus* populations; Table S2: Details of the species used to detect the transferability of molecular markers in this study; Table S3: List of the 58 primer pairs with expected size of PCR products; Table S4: Detailed information of EST-SSRs based on the number of nucleotide repeat units.

## References

[1] Dewey D.R., Gustafson J.P. (1984). The Genomic System of Classification as a Guide to Intergeneric Hybridization with the Perennial Triticeae.

[2] Löve À. (1984). Conspectus of the Triticeae. Feddes Repert..

[3] Cainong J.C., Bockus W.W., Feng Y., Chen P., Qi L., Sehgal S.K., Danilova T.V., Koo D., Friebe B., Gill B.S. (2015). Chromosome engineering, mapping, and transferring of resistance to Fusarium head blight disease from *Elymus tsukushiensis* into wheat. Theor. Appl. Genet..

[4] Keng Y.L. (1959). Flora Illustralis Plantarum Primarum Sinicarum, Gramineae.

[5] Chen S.L., Zhu G.H., Wu Z.Y., Raven P.H., Hong D.Y. (2006). Tribus Triticeae, Poaceae.

[6] Muriira N.G., Muchugi A., Yu A., Xu J., Liu A. (2018). Genetic diversity analysis reveals genetic differentiation and strong population structure in *Calotropis* plants. Sci. Rep..

[7] Khan F. (2015). Molecular markers: An excellent tool for genetic analysis. J. Mol. Biomark. Diagn..

[8] Arif I.A., Khan H.A., Bahkali A.H., Al Homaidan A.A., Al Farhan A.H., Al Sadoon M., Shobrak M. (2011). DNA marker technology for wildlife conservation. Saudi J. Biol. Sci..

[9] Lee H.J., Park H.R., Lee A.J., Da E.N., Chung K.W. (2020). Genetic authentication of cultivars with flower variant types using SSR markers in spring orchid, *Cymbidium goeringii*. Hortic. Environ. Biotechnol..

[10] Chikh-Rouhou H., Mezghani N., Mnasri S., Mezghani N., Garcés-Claver A. (2021). Assessing the Genetic Diversity and Population Structure of a Tunisian Melon (*Cucumis melo* L.) Collection Using Phenotypic Traits and SSR Molecular Markers. Agronomy.

[11] Sun M., Dong Z.X., Yang J., Wu W.D., Zhang C.L., Zhang J.B., Xiong Y., Jia S.G., Ma X. (2021). Transcriptomic resources for prairie grass (*Bromus catharticus*): Expressed transcripts, tissue-specific genes and identification and validation of EST-SSR markers. BMC Plant Biol..

[12] Song Y.P., Jiang X.B., Zhang M., Wang Z.L., Bo W.H., An X.M., Zhang Z.Y. (2012). Differences of EST-SSR and genomic-SSR markers in assessing genetic diversity in poplar. For. Stud. China.

[13] Gu X.Y., Guo Z.H., Ma X., Bai S.Q., Zhang X.Q., Zhang C.B., Chen S.Y., Peng Y., Yan Y.H., Huang L.K. (2015). Population genetic variability and structure of *Elymus breviaristatus* (Poaceae: Triticeae) endemic to Qinghai–Tibetan Plateau inferred from SSR markers. Biochem. Syst. Ecol..

[14] Yu Q.Q., Liu Q., Xiong Y., Xiong Y.L., Dong Z.X., Yang J., Liu W., Ma X., Bai S.Q. (2019). Genetic diversity and population divergence of a rare, endemic grass (*Elymus breviaristatus*) in the southeastern Qinghai-Tibetan Plateau. Sustainability.

[15] Zhang Z.Y., Xie W.G., Zhao Y.Q., Zhang J.C., Wang N., Ntakirutimana F., Yan J.J., Wang Y.R. (2019). EST-SSR marker development based on RNA-sequencing of *E. sibiricus* and its application for phylogenetic relationships analysis of seventeen *Elymus* species. BMC Plant Biol..

[16] Xiong Y., Lei X., Bai S.Q., Xiong Y.L., Liu W.H., Wu W.D., Yu Q.Q., Dong Z.X., Yang J., Ma X. (2021). Genomic survey sequencing, development and characterization of single- and multi-locus genomic SSR markers of *Elymus sibiricus* L.. BMC Plant Biol..

[17] Taheri S., Abdullah T.L., Rafii M.Y., Harikrishna J.A., Werbrouck S.P.O., Teo C.H., Sahebi M., Azizi P. (2019). De novo assembly of transcriptomes, mining, and development of novel EST-SSR markers in *Curcuma alismatifolia* (Zingiberaceae family) through Illumina sequencing. Sci. Rep..

[18] Karan M., Evans D.S., Reilly D., Schulte K., Wright C., Innes D., Holton T.A., Nikles D.G., Dickinson G.R. (2012). Rapid microsatellite marker development for African mahogany (*Khaya senegalensis*, Meliaceae) using next-generation sequencing and assessment of its intra-specific genetic diversity. Mol. Ecol. Resour..

[19] Grabherr M.G., Haas B.J., Yassour M., Levin J.Z., Thompson D.A., Amit I., Adiconis X., Fan L., Raychowdhury R., Zeng Q.D. (2011). Full-length transcriptome assembly from RNA Seq data without a reference genome. Nat. Biotechnol..

[20] Ashburner M., Ball C.A., Blake J.A., Botstein D., Butler H., Cherry J.M., Davis A.P., Dolinski K., Dwight S.S., Eppig J.T. (2000). Gene ontology: Tool for the unification of biology. Nat. Genet..

[21] Tatusov R.L., Galperin M.Y., Natale D.A. (2000). The COG database: A tool for genome scale analysis of protein functions and evolution. Nucleic Acids Res..

[22] Koonin E.V., Fedorova N.D., Jackson J.D., Jacobs A.R., Krylov D.M., Makarova K.S., Mazumder R., Mekhedov S.L., Nikolskaya A.N., Rao B.S. (2004). A comprehensive evolutionary classification of proteins encoded in complete eukaryotic genomes. Genome Biol..

[23] Kanehisa M., Goto S., Kawashima S., Okuno Y., Hattori M. (2004). The KEGG resource for deciphering the genome. Nucleic Acids Res..

[24] Deng Y.Y., Li J.Q., Wu S.F., Zhu Y.P., Chen Y.W., He F.C. (2006). Integrated nr database in protein annotation system and its localization. Comput. Eng..

[25] Finn R.D., Bateman A., Clements J., Coggill P., Eberhardt R.Y., Eddy S.R., Heger A., Hetherington K., Holm L., Mistry J. (2014). Pfam: The protein families database. Nucleic Acids Res..

[26] Thiel T., Michalek W., Varshney R., Graner A. (2003). Exploiting EST databases for the development and characterization of gene-derived SSR-markers in barley (*Hordeum vulgare* L.). Theor. Appl. Genet..

[27] Yeh F.C. (1997). Population genetic analysis of codominant and dominant markers and quantitative traits. Belg. J. Bot..

[28] Roldán-Ruiz I., Dendauw J., Bockstaele E.V., Depicker A., Loose M.D. (2000). AFLP markers reveal high polymorphic rates in ryegrasses (*Lolium* spp.). Mol. Breed..

[29] Rohlf F.J. (1987). NTSYS-pc: Microcomputer programs for numerical taxonomy and multivariate analysis. Am. Stat..

[30] Tamura K., Stecher G., Peterson D., Filipski A., Kumar S. (2013). MEGA6: Molecular Evolutionary Genetics Analysis Version 6.0. Mol. Boil. Evol..

[31] Peakall R., Smouse P.E. (2012). GenAlEx 6.5: Genetic analysis in Excel. Population genetic software for teaching and research—An update. Bioinformatics.

[32] Falush D., Stephens M., Pritchard J.K. (2007). Inference of population structure using multilocus genotype data: Dominant markers and null alleles. Mol. Ecol. Notes.

[33] Earl D.A., Vonholdt B.M. (2012). Structure Harvester: A website and program for visualizing STRUCTURE output and implementing the Evanno method. Conserv. Genet. Resour..

[34] Kopelman N.M., Mayzel J., Jakobsson M., Rosenberg N.A., Mayrose I. (2015). Clumpak: A program for identifying clustering modes and packaging population structure inferences across K. Mol. Ecol. Resour..

[35] Bushman B.S., Larson S.R., Mott I.W., Cliften P.F., Wang R.R.C., Chatterton N.J., Hernandez A.G., Ali S., Kim R.W., Thimmapuram J. (2008). Development and annotation of perennial Triticeae ESTs and SSR markers. Genome.

[36] Mott I.W., Larson S.R., Bushman B.S. (2011). Simple sequence repeat (SSR) markers for *Elymus*, *Pseudoroegneria* and *Pascopyrum* species (*Triticeae: Gramineae*). Plant Genet. Resour..

[37] Sun G.L., Salomon B., Von B.R. (1998). Characterization of microsatellite loci from *Elymus alaskanus* and length polymorphism in several *Elymus* species (*Triticeae: Poaceae*). Genome.

[38] Liu Y.L., Zhang P.F., Song M.L., Hou J.L., Qing M., Wang W.Q., Liu C.S., Chen S.L. (2015). Transcriptome analysis and development of SSR molecular markers in *Glycyrrhiza uralensis* fisch. PLoS ONE.

[39] Wang S.F., Wang X.F., He Q.W., Liu X.X., Xu W.L., Li L.B., Gao J.W., Wang F.D. (2012). Transcriptome analysis of the roots at early and late seedling stages using Illumina paired-end sequencing and development of EST-SSR markers in radish. Plant Cell Rep..

[40] Hiz M.C., Canher B., Niron H., Turet M. (2014). Transcriptome analysis of salt tolerant common bean (*Phaseolus vulgaris* L.) under saline conditions. PLoS ONE..

[41] Luo D., Zhou Q., Ma L.C., Xie W.G., Wang Y.R., Hu X.W., Liu Z.P. (2015). Novel polymorphic expressed–sequence tag–simple–sequence repeat markers in *Campeiostachys nutans* for genetic diversity analyses. Crop Sci..

[42] Wang Z., Yan H.W., Fu X.N., Li X.H., Gao H.W. (2013). Development of simple sequence repeat markers and diversity analysis in alfalfa (*Medicago sativa* L.). Mol. Biol. Rep..

[43] Xin D.W., Sun J.Y., Wang J.L., Jiang H.W., Hu G.H., Liu C. (2012). Identification and characterization of SSRs from soybean (*Glycine max*) ESTs. Mol. Biol. Rep..

[44] Eujayl I., Sledge M.K., Wang L., May G.D., Chekhovskiy K., Zwonitzer J.C., Mian M.A.R. (2004). *Medicago truncatula* EST-SSRs reveal cross-species geneticmarkers for *Medicago* spp.. Theor. Appl. Genet..

[45] Wang Y., Pan Y., Liu Z., Zhu X.W., Zhai L.L., Xu L., Yu R.G., Gong Y.Q., Liu L.W. (2013). De novo transcriptome sequencing of radish (*Raphanus sativus* L.) and analysis of major genes involved in glucosinolate metabolism. BMC Genom..

[46] Hina F., Yisilam G., Wang S.Y., Li P., Fu C.X. (2020). De novo transcriptome assembly, gene annotation and SSR marker development in the moon seed genus *Menispermum* (Menispermaceae). Front. Genet..

[47] Varshney R.K., Thiel T., Stein N., Langridge P., Graner A. (2002). In silico analysis on frequency and distribution of microsatellites in ESTs of some cereal species. Cell. Mol. Biol. Lett..

[48] You Y.N., Liu D.C., Liu H.B., Zheng X.F., Diao Y., Huang X.F., Hu Z.L. (2015). Development and characterisation of EST-SSR markers by transcriptome sequencing in taro (*Colocasia esculenta* (L.) Schoot). Mol. Breed..

[49] Wu J., Cai C.F., Cheng F.Y., Cui H.L., Zhou H. (2014). Characterisation and development of EST-SSR markers in tree peony using transcriptome sequences. Mol. Breed..

[50] Kuleung C., Baenziger P.S., Dweikat I. (2004). Transferability of SSR markers among wheat, rye, and triticale. Theor. Appl. Genet..

[51] Gitzendanner M.A., Soltis P.S. (2000). Patterns of genetic variation in rare and widespread plant congeners. Am. J. Bot..

[52] Sanders T.B., Hamrick J.L., Holden L.R. (1979). Allozyme variation in *Elymus canadensis* from the tallgrass prairie region: Geographic variation. Am. Midl. Nat..

[53] Díaz O., Sun G.L., Salomon B., Von Bothmer R. (2000). Levels and distribution of allozyme and RAPD variation in populations of *Elymus fibrosus* (Schrenk) Tzvel. (Poaceae). Genet. Resour. Crop Evol..

[54] Sun G.L., Diaz O., Salomon B., Von Bothmer R. (2001). Genetic diversity and structure in a natural *Elymus caninus* population from Denmark based on microsatellite and isozyme analyses. Plant Syst. Evol..

[55] Yan X.B., Guo Y.X., Zhou H., Lu B.R., Wang K. (2006). Genetic patterns of ten *Elymus* species from the Tibetan and Inner Mongolian plateaus of China. Grass Forage Sci..

[56] Brzosko E., Ratkiewicz M., Wróblewska A. (2015). Allozyme differentiation and genetic structure of the Lady’s slipper (*Cypripedium calceolus*) island populations in north-east Poland. Bot. J. Linn. Soc..

[57] Zhou Q., Luo D., Ma L.C., Xie W.G., Wang Y., Wang Y.R., Liu Z.P. (2016). Development and cross-species transferability of EST-SSR markers in Siberian wildrye (*Elymus sibiricus* L.) using Illumina sequencing. Sci. Rep..

[58] Chen S.Y., Ma X., Zhang X.Q., Chen Z.H. (2008). Karyotypes of 10 tetraploid species in *Elymus* (Poaceae: Triticeae). J. Syst. Evol..

[59] Baum B.R., Yen C., Yang J.L. (1995). Taxonomic separation of *Kengyilia* (Poaceae: Triticeae) in relation to nearest related *Roegneria*, *Elymus*, and *Agropyron*, based on some morphological characters. Plant Syst. Evol..

