# Supplementary material for: Characterization and Application of EST-SSR Markers Developed from Transcriptome Sequences in Elymus breviaristatus (Poaceae: Triticeae)
Source: Genes (Basel). 2023 Jan 23;14(2):302. doi: 10.3390/genes14020302 (PMC9957396; doi:10.3390/genes14020302)
Supplement: Supplementary file 1 [file genes-14-00302-s001.zip › genes-2129890 - Supplementary.pdf]

Table S1. Geographic information of the 12 E. breviaristatus populations.

Pop ID	Pop Size	Collection Site	Latitude	Longitude	Altitude (m)
Pop1	15	Zhubalong, Markam Country, Qamdo Prefecture	29°44'11.950″	98°43'23.122″	3530
Pop2	15	Wangda, Zogang Country, Qamdo Prefecture	29°42'27.821″	97°47'29.187″	3840
Pop3	14	Jida, Baxoi Country, Qamdo Prefecture	29°59'01.579″	96°40'13.665″	3776
Pop4	15	Ranwu, Baxoi Country, Qamdo Prefecture	29°32'26.570″	96°46'40.956″	3820
Pop5	15	Zhaxigang,Maizhokunggar Country, Lhasa Prefecture	29°44'14.377″	90°49'25.706″	4025
Pop6	15	Yala, Sog Country, Nagqu Prefecture	31°49'35.868″	93°33'43.773″	4071
Pop7	15	Bada, Dengqen Country, Qamdo Prefecture	31°41'18.650″	94°53'56.544″	4525
Pop8	15	Bingda, Riwoqe Country, Qamdo Prefecture	31°07'09.215″	96°47'34.984″	3708
Pop9	15	Chaiwei, Karuo District, Qamdo Prefecture	31°28'50.879″	97°14'19.888″	3392
Pop10	15	Qingnidong, Jomda Country, Qamdo Prefecture	31°19'28.580″	97°56'16.266″	3952
Pop11	15	Jiaxing, Gongbo'gyamda Country, Nyingchi Prefecture	29°52'47.348″	92°42'05.058″	3969
Pop12	15	Tiantuo, Zogang Country, Qamdo Prefecture	29°51'06.854″	97°41'04.325″	3909

Table S2. Details of the species used to detect the transferability of molecular markers in this study.

Species	Genome	Accessions PI	Origins
E. dahuricus	StHY	PI639853	Xizang, China
E. dahuricus var. violeus	StHY	HY-3	Sichuan, China
E. atratus	StHY	HY-1	Sichuan, China
E. cylindricus	StHY	HY-4	Sichuan, China
E. excelsus	StHY	G05449	Inner Mongolia, China
E. purpuraristatus	StHY	KD-1	Sichuan, China
E. himalayanus	StHY	H4134	Pakistan
E. drobovii	StHY	H10299	Iran
E. nutans	StHY	Y2196	Xizang, China
E. breviaristatus	StHY	PI504447	China
E. submuticus	StHY	PI531684	China
E. tangutorum	StHY	HY-1	Sichuan, China
R. kamoji	StHY	R273	Sichuan, China
E. tsukushiensis	StHY	PI499623	Japan
E. humidus	StHY	J3279S	Japan
E. schrenkianus	StHY	PI659920	Kyrgyzstan
R. aristiglumis	StHY	PI598486	Xinjiang,China
E. tschimganicus	StStY	W614438	Kazakhstan
E. glaucissimus	StStY	PI611142	Kazakhstan
E. transhyrcanus	StStH	PI383579	Ukraine
E. repens	StStH	PI565006	Russian Federation
E. patagonicus	StHH	PI297898	Argentina
E. scabriglumis	StHH	PI331168	Argentina

Table S3. Detailed information of EST-SSRs based on the number of nucleotide repeat unit.

Repeats	5	6	7	8	9	10	11	12	13	14	15	16	17	18	19	20	＞20	Total
A/T	-	-	-	-	-	3469	1337	747	359	319	240	231	129	66	67	40	760	7764
C/G	-	-	-	-	-	147	123	93	122	83	92	51	51	39	36	57	438	1332
AC/GT	-	559	220	223	97	89	93	83	20	45	38	27	18	15	34	21	45	1627
AG/CT	-	1173	563	369	396	170	171	209	67	71	85	76	59	41	38	23	91	3602
AT/AT	-	242	132	61	43	26	36	15	20	10	15	7	9	7	6	6	26	661
CG/CG	-	289	46	15	1												0	351
AAC/GTT	140	66	40	46	10	4	6		3								0	315
AAG/CTT	614	204	90	58	6	18	13	7	14	3	1						0	1028
AAT/ATT	59	37	9	15	3	11			2						1		0	137
ACC/GGT	490	232	60	14	2	10			1								0	809
ACG/CGT	503	165	50	17	4	3											0	742
ACT/AGT	81	42	12	4	9										5		0	153
AGC/CTG	1349	584	189	99	16	15	2	1					1	2			0	2258
AGG/CCT	1533	660	214	106	9	16	3	5	2								0	2548
ATC/ATG	377	118	68	22	4	11		1		2							0	603
CCG/CGG	3541	1436	628	179	25	8	3	3									0	5823
AAAC/GTTT	7	2															0	9
AAAG/CTTT	28	12	3	1	1												0	45
AAAT/ATTT	11	1															0	12
AACC/GGTT	14	3															0	17
AACG/CGTT	1	1															0	2
AAGC/CTTG	22	2															0	24
AAGG/CCTT	43	18	1														0	62
AATC/ATTG	10	8															0	18
AATG/ATTC	8	9	2														0	19
AATT/AATT	1																0	1
ACAG/CTGT	4																0	4
ACAT/ATGT	17	21	1	9													0	48
ACCC/GGGT	1																0	1
ACCG/CGGT	10																0	10
ACCT/AGGT	4	3															0	7
ACGC/CGTG	57	11															0	68
ACGG/CCGT	14	2	2														0	18
ACGT/ACGT	7																0	7
ACTC/AGTG	1	2															0	3
ACTG/AGTC	6	1															0	7
AGAT/ATCT	5	10	1		2		1	2									0	21
AGCC/CTGG	18	4															0	22
AGCG/CGCT	23	7															0	30
AGCT/AGCT	6																0	6
AGGC/CCTG	41	18															0	59
AGGG/CCCT	51	17															0	68
ATCC/ATGG	28	8	12														0	48
ATCG/ATCG	2																0	2
ATGC/ATGC	10																0	10
CCCG/CGGG	44	6		2													0	52
CCGG/CCGG	11																0	11

AAAAC/GTTTT	6				0	6
AAAAG/CTTTT	3				0	3
AAAAT/ATTTT	2				0	2
AAACC/GGTTT	3	1			0	4
AAAGG/CCTTT	2				0	2
AAATC/ATTTG	5				0	5
AACAC/GTGTT	1				0	1
AACCC/GGGTT	1				0	1
AACTC/AGTTG		1			0	1
AACTG/AGTTC	3				0	3
AAGAG/CTCTT	8		4		0	12
AAGCC/CTTGG	5				0	5
AAGCT/AGCTT	1				0	1
AAGGG/CCCTT	12				0	12
AATAC/ATTGT			2	1	0	3
AATCT/AGATT	2				0	2
ACAGC/CTGTG	1				0	1
ACATC/ATGTG	2				0	2
ACCCC/GGGGT	6				0	6
ACCCG/CGGGT	1				0	1
ACCCT/AGGGT	6				0	6
ACCTC/AGGTG	1				0	1
ACCTG/AGGTC	2				0	2
ACGAG/CGTCT	2				0	2
ACGCC/CGTGG	2				0	2
ACGGG/CCCGT	1				0	1
ACTCC/AGTGG	1				0	1
AGAGC/CTCTG	5				0	5
AGAGG/CCTCT	5	2			0	7
AGATG/ATCTC	2				0	2
AGCCC/CTGGG	1				0	1
AGCGC/CGCTG			1		0	1
AGCGG/CCGCT	1				0	1
AGCTC/AGCTG	3				0	3
AGGCG/CCTCG	1				0	1
AGGGC/CCCTG	8				0	8
AGGGG/CCCCT	2				0	2
ATATC/ATATG		1			0	1
ATCCC/ATGGG	7				0	7
ATGCC/ATGGC	1				0	1
CCCCG/CGGGG	4				0	4
AAAATG/ATTTTC		1			0	1
AAAGCC/CTTTGG	3				0	3
AACGGC/CCGTTG	2				0	2
AAGAGG/CCTCTT	2	1			0	3

AAGCCG/CGGCTT	1			0	1
AAGGAG/CCTTCT	1			0	1
AATGGC/ATTGCC	1			0	1
AATGTG/ACATTC	2			0	2
ACACAT/ATGTGT	1	2		0	3
ACACGC/CGTGTG		4		0	4
ACAGGC/CCTGTG	1			0	1
ACCAGC/CTGGTG	4			0	4
ACCTCC/AGGTGG	3	2		0	5
ACCTCG/AGGTCG	1			0	1
ACGAGG/CCTCGT		1		0	1
ACGCAG/CGTCTG	3			0	3
ACGGCG/CCGTCG		3		0	3
AGAGGC/CCTCTG			1	0	1
AGATGG/ATCTCC	3			0	3
AGCAGG/CCTGCT	7	2		0	9
AGCGGC/CCGCTG		1		0	1
AGCTCC/AGCTGG	1			0	1
AGGATG/ATCCTC	2			0	2
AGGCGG/CCGCCT	7			0	7
ATCCCC/ATGGGG	2			0	2
ATCGCC/ATGGCG	1			0	1
ATCGGC/ATGCCG	5			0	5
CCCCCG/CGGGGG	1			0	1

Table S4. List of the 58 primer pairs with expected size of PCR products.

Primers	Repeat Motif	Forward Primer (5'-3')	Tm (°C)	Reverse Primer (5'-3')	Tm (°C)	Note
MDEB002	(CT)9	AGTCCTTAGCCCCTAGGACG	60.106	AAAGAGAGGAGGGCTGGGAT	59.955	Polymorphic
MDEB024	(CAT)5	ATGTATGGGGCGGGCATATG	60.034	CAAACCGACGAGAGGTGTCA	59.968	Polymorphic
MDEB025	(CCA)5	GATCCGACGACCTCAGCTTC	60.249	CCATCTGCAGCTGGTCAAGA	60.036	Polymorphic
MDEB027	(CCG)5	CATCGACATCACCTGGGTGT	59.75	TTCTTGTTCCCCACGGCTTC	60.536	Polymorphic
MDEB046	(CTA)6	TACATCAAAGCGCAGGCAGA	60.036	AAGGAGTTGACATGGCTCGG	60.036	Polymorphic
MDEB050	(CTG)5	CGCCTACACAAGATGGTGGT	60.036	CACTCTGTCGAAATGCAGCG	59.906	Polymorphic
MDEB051	(CTG)5	GCCTGCTGAAGATGCTCAGA	60.108	CCAGCAGTACAACCAGAGCA	59.965	Polymorphic
MDEB054	(GAC)6	ACCGGCATTTCCTACACTGG	60.035	TCCTCACAGCTCTCACCACA	60.47	Polymorphic
MDEB063	(GCG)5	CACTTCTCCCCTCGCGATG	60.227	AACATGAGCAGGCTCTCGAC	60.108	Polymorphic
MDEB065	(GCG)5	CAAGAGCGACCTGGTGAAGT	59.966	CTCGTTCCCATCATCCGCAA	60.462	Polymorphic
MDEB067	(GCG)6	AATGGTTCGTTGTCGTCGGA	59.968	GTGGTGGACAAGTAGCTGCT	59.965	Polymorphic
MDEB068	(GCT)5	GTAACTGTTCTTCGGCGGGA	60.038	CCACCGAAGAACGACTACCC	60.109	Polymorphic
MDEB076	(GGC)5	ATCCACGTTTCCCTCTGCTG	60.036	CCAGTCCCCACTACCAGCTA	60.325	Polymorphic
MDEB083	(GTC)5	TGAGGCTGGGACTGAAGAGT	60.179	CTTCTCAGCTGCTGACCTCC	60.108	Polymorphic
MDEB091	(TCA)6	ACCCCCAGCTACTACCATGT	59.956	GAGATGGAGCCTACCGAAGC	59.967	Polymorphic
MDEB094	(TCG)5	CGCCTCTTCCACGTCTTTGA	60.32	GATGGTGCTCCTCGAGATCG	60.04	Polymorphic
MDEB095	(TGT)5	CAGCTGTTCTGGAGGGTGAG	59.896	TCAGAAGGTGACGACGCTTC	59.969	Polymorphic
MDEB100	(TGT)5	TGGTCGCGAGATCTTATGGG	59.326	TAAACAACAGCACCTGGCCT	59.815	Polymorphic
MDEB003	(GA)9	AATCGACATGAGAGCACCGG	60.179	CGGCCCTTTTTATCCCCTGT	60.034	Monomorphic
MDEB004	(AAC)6	TTCAGGCATCCCGCTAACAG	60.108	TCCTACCTCCAAGCCCTCTT	59.582	Monomorphic
MDEB009	(ACG)6	GGCCTCTACGAGTTCCCCTA	60.106	GGCACACCCTACACGTACTC	60.109	Monomorphic
MDEB010	(ACT)5	AGGAACGCATAGGCACACTC	60.108	ATTCACCCCGTGATCGATCG	59.968	Monomorphic
MDEB012	(AGC)5	CGTCGTGGAAGCTGAGGTAG	60.179	CTTGCAAGTTACTTCCGCCG	59.834	Monomorphic
MDEB014	(AGG)6	TTCTTGAACGCCACCTCAGG	60.25	AGAGAGAAGCCAAGAACCGC	60.037	Monomorphic

MDEB017	(ATC)5	TCTTTGACCAGCTGCGAAGT	59.893	CTGGCCGAGAACAAGATCGT	60.109	Monomorphic
MDEB020	(CAA)5	CAGTTCAGTAGCAGCCACCA	59.965	TGGGCGTCAAGTTTGGATGA	59.89	Monomorphic
MDEB021	(CAC)5	CAGGATCTTCCAGCTCACCC	59.82	GGAGGAGTTCATGAAGCCGG	60.463	Monomorphic
MDEB022	(CAG)5	CAATCGACAAGCTCCCCGAG	60.808	GGCCTCCTTGTCCTTCACAG	60.322	Monomorphic
MDEB031	(CCT)5	GACGGTCTGGTCGAACACTT	59.968	GGAATCCCCTTTGACTCCCG	60.107	Monomorphic
MDEB032	(CCT)6	CCTCCGGCTTCAAGCTCTTC	60.743	GAACTTGGTCTCCCTGCTCC	60.036	Monomorphic
MDEB033	(CGA)5	GCAACACGAACCGGAAATGA	59.411	TGTCTGTACATAGCTGCGGC	60.179	Monomorphic
MDEB034	(CGA)6	CCTCGCCGCTGGACTACA	61.449	TTCTTCCGTTGCCTCTGACC	59.965	Monomorphic
MDEB036	(CGC)5	GCAGTACTACCAGCAGGGAC	59.824	GAGATCTCCGATCCACAGCG	60.04	Monomorphic
MDEB037	(CGC)5	CCATCACGTGATCTCCGAGG	59.969	CCTCTCGATCCAACTCGACG	59.972	Monomorphic
MDEB041	(CGG)5	TCCACTCTGTCTCGTCGACT	59.965	GCTGTTGCCACCTCTGATGA	60.322	Monomorphic
MDEB043	(CGG)6	CGCCGACAAGAACCGAGATT	60.736	GGACGGGGAAAATGGGGATT	60.033	Monomorphic
MDEB047	(CTC)5	GCGACTGGATTAGATGGCGA	59.968	TTCTACGCACCTTCACCGTG	60.319	Monomorphic
MDEB048	(CTC)5	CGGAAGCTCATCGAGATGCT	59.968	TGACCCTCCTCAGCAGATCA	59.958	Monomorphic
MDEB049	(CTC)6	CGTCATGCCCTTGAACTCCT	60.036	AAGACCAGAAGCAGAAGGGC	59.963	Monomorphic
MDEB053	(GAA)5	TTGAGGTCAGTGGCAAGCAA	60.107	GTTTTCGCGGTCTTGACGAG	59.839	Monomorphic
MDEB066	(GCG)6	GGACGGCCATGCATAACAGG	61.447	GAGCAGCTGGTCCATCATCG	60.879	Monomorphic
MDEB069	(GCT)5	TCCACCATGCTCAGCGAATT	60.035	TCTCGGTAAACGGTCCTTGC	60.038	Monomorphic
MDEB070	(GCT)6	CTCCGCATCTTTTTCAGCGG	59.902	CGGCCGGGTTAAATTCAACC	59.828	Monomorphic
MDEB071	(GGA)5	GGAGAGGTGGAGTCGTCGTC	61.647	TTCCTGTCCTTCACCAACCC	59.523	Monomorphic
MDEB072	(GGA)5	CCGCCGCCATGATGAAGTAT	60.604	ACCGACTCCTCCTCGTACTC	60.108	Monomorphic
MDEB078	(GGC)6	GCGAAAGGGAGGGAATGTCA	60.035	CTCCCCAACTTCCCCTCCTA	59.956	Monomorphic
MDEB082	(GTA)5	GAGCAGTCCAGGCCATTGAT	60.107	TCACCACCTCCTCCTCCAAG	60.545	Monomorphic
MDEB084	(GTC)6	GGACGCAGAGAAGGAGTACG	59.901	TGTCCGTATGGCAGCGATAC	59.968	Monomorphic
MDEB085	(GTG)5	ACAGATGACACGCGGTACAG	60.109	TCGCCAAACCTGTCTGATCC	60.036	Monomorphic

MDEB087	(GTT)5	ATCACACAAGCCAGAGGAGC	60.036	GCTCCAGTACAACAAACCGC	59.763	Monomorphic
MDEB088	(TAC)5	GCCTCCTCCGCCTCTAGTAT	60.251	TCATGGCTGGACCCCATCTA	60.03	Monomorphic
MDEB089	(TAG)5	AAGCAGCGGGTATGCAAAAC	59.756	CAAGTCGCACCAAGAACAGC	60.041	Monomorphic
MDEB090	(TAT)5	AGGTGGTTGCTATTGGCTCC	60.034	GCTGTTAGATATTTTGGCGCCC	60.288	Monomorphic
MDEB092	(TCC)5	GCAAAACGAGTGTGACTGGC	60.317	AGAATTCCGGCGCTTCTTCA	60.036	Monomorphic
MDEB093	(TCC)6	GGTGAAGCTCTAGACCCCCA	60.618	CAAGCTCAAGGACCACGACT	59.966	Monomorphic
MDEB098	(TGC)6	CACATTGTCACGCTGCCAAA	59.969	CGAATGCCTTTGGACCGTTG	60.11	Monomorphic
MDEB099	(TGG)5	CTCACTCGCTATGCAGCTCG	60.934	GAGCTCCAGCACCTCCATAG	59.608	Monomorphic
MDEB101	(TTA)5	GCGACATGGGCAAGAACAAG	60.11	GTCCCGCAAACAAGTCACAC	59.971	Monomorphic
